# Survival Analysis Part II: Multivariate data analysis – an introduction to concepts and methods

**DOI:** 10.1038/sj.bjc.6601119

**Published:** 2003-07-29

**Authors:** M J Bradburn, T G Clark, S B Love, D G Altman

**Affiliations:** 1 Cancer Research UK/NHS Centre for Statistics in Medicine, Institute of Health Sciences, Old Road, Oxford OX3 7LF, UK

**Keywords:** survival analysis, Cox model, AFT model, model selection

## INTRODUCTION

Survival analysis involves the consideration of the time between a fixed starting point (e.g. diagnosis of cancer) and a terminating event (e.g. death). The key feature that distinguishes such data from other types is that the event will not necessarily have occurred in all individuals by the time the study ends, and for these patients, their full survival times are unknown. For instance, in studies that measure the length of survival after diagnosis of cancer, it is common for a proportion of individuals to remain alive and disease-free at the end of the follow-up period, and for these patients, we know only a lower limit on their actual time to event. Thus, special methods are required for these type of data. The explanation and demonstration of some of the methods proposed to analyse such data are the basis of this series.

In the first paper of this series (Clark *et al*, 2003), we described initial methods for analysing and summarising survival data including the definition of hazard and survival functions, and testing for a difference between two groups. We continue here by considering various statistical models and, in particular, how to estimate the effect of one or more factors that may predict survival.

## THE NEED FOR MULTIVARIATE STATISTICAL MODELLING

The previous paper demonstrated the construction of (Kaplan–Meier) survival curves for different patient groups, and introduced the logrank test to investigate differences between them. Both these methods are examples of *univariate* analysis; they describe the survival with respect to the factor under investigation, but necessarily ignore the impact of any others. It is more common, at least in clinical investigations, to have a situation where several (known) quantities or *covariates*, potentially affect patient prognosis. For example, suppose two groups of patients are compared: those with and those without a specific genotype. If one of the groups also contains older individuals, any difference in survival may be attributable to genotype or age or indeed both. Hence, when investigating survival in relation to any one factor, it is often desirable to adjust for the impact of others. Moreover, while the logrank test provides a *P*-value for the differences between the groups, it offers no estimate of the actual effect size; in other words, it offers a statistical, but not a clinical, assessment of the factor's impact. The use of a statistical model improves on these methods by allowing survival to be assessed with respect to several factors simultaneously, and in addition, offers estimates of the strength of effect for each constituent factor. Therefore, statistical models are important and frequently used tools which, when constructed appropriately, offer valuable insight into the survival process.

Several statistical methods have been proposed for modelling survival analysis data. We will describe the most important models and illustrate their application using example datasets described in the previous paper (Clark *et al*, 2003). As before, we will assume throughout that all survival times are independent of each other and that censoring occurs solely as right-censoring and is uninformative. The focus is on covariates that are measured at the time of entry to the study, that may be continuous (e.g. the patient age or tumour size), binary (e.g. gender), unordered categorical (e.g. histology) or ordered categorical or ordinal (e.g. performance status or FIGO stage). In the next paper in this series, we will discuss the statistical assumptions made when using statistical models, and provide advice on choosing the appropriate model and covariates therein. We will also consider how to model covariates that change values over time (called ‘time-dependent’ or ‘updated’ covariates).

The methods we present here may be divided into two broad categories: proportional hazard approaches (including the semiparametric Cox model and fully parametric approaches) and accelerated failure time models. These methods have different properties and interpretations, but all may be used to summarise survival data.

## THE COX (‘SEMI-PARAMETRIC’) PROPORTIONAL HAZARDS MODEL

The Cox (proportional hazards or PH) model (Cox, 1972) is the most commonly used multivariate approach for analysing survival time data in medical research. It is a survival analysis regression model, which describes the relation between the event incidence, as expressed by the hazard function and a set of covariates. A fuller explanation of the hazard function was given in the previous article (Clark *et al*, 2003). Put briefly, the hazard is the instantaneous event probability at a given time, or the probability that an individual under observation experiences the event in a period centred around that point in time.

Mathematically, the Cox model is written as

*h*(*t*)=*h*_0_(*t*) × exp{*b*_1_*x*_1_+*b*_2_*x*_2_+⋯+*b*_*p*_*x*_*p*_}

where the hazard function *h*(*t*) is dependent on (or determined by) a set of *p* covariates (*x*_1_, *x*_2_, …, *x_p_*), whose impact is measured by the size of the respective *coefficients* (*b*_1_, *b*_2_, …, *b_p_*). The term *h*_0_ is called the baseline hazard, and is the value of the hazard if all the *x_i_* are equal to zero (the quantity exp(0) equals 1). The ‘*t*’ in *h*(*t*) reminds us that the hazard may (and probably will) vary over time. An appealing feature of the Cox model is that the baseline hazard function is estimated nonparametrically, and so unlike most other statistical models, the survival times are not assumed to follow a particular statistical distribution.

**Figure 1:**
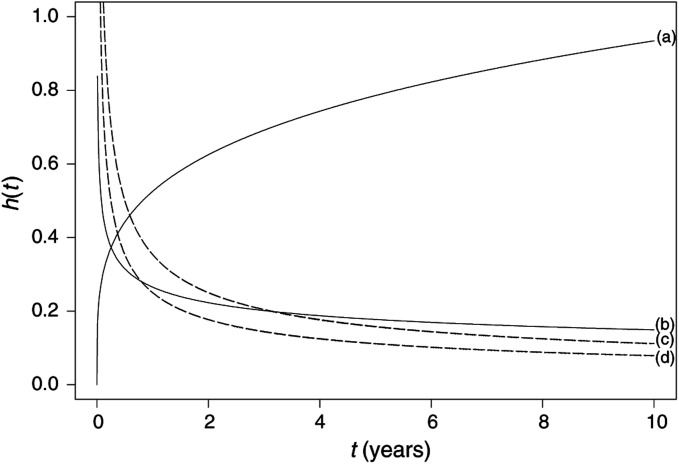
Example of (non-) proportional hazards (groups (c) and (d) only have proportional hazards) using the Weibull distribution. For the Weibull survival model, the hazard function *h*(*t*)=*λs*(*λt*)^*s*−1^ for *λ*, *s*>0: (a) increasing hazard (*λ*=0.5, *s*=1.25); (b) decreasing hazard (*λ*=0.25, *s*=0.75); (c) decreasing hazard (*λ*=0.5, *s*=0.5); (d) decreasing hazard (*λ*=0.25, *s*=0.5).

The Cox model is essentially a multiple linear regression of the logarithm of the hazard on the variables *x_i_*, with the baseline hazard being an ‘intercept’ term that varies with time. The covariates then act multiplicatively on the hazard at any point in time, and this provides us with the key assumption of the PH model: the hazard of the event in any group is a constant multiple of the hazard in any other. This assumption implies that the hazard curves for the groups should be proportional and cannot cross (see Figure 1

for examples of each). Proportionality implies that the quantities exp(*b_i_*) are called *hazard ratios*. A value of *b_i_* greater than zero, or equivalently a hazard ratio greater than one, indicates that as the value of the *i*th covariate increases, the event hazard increases and thus the length of survival decreases. Put another way, a hazard ratio above 1 indicates a covariate that is positively associated with the event probability, and thus negatively associated with the length of survival. This *proportionality assumption* is often appropriate for survival time data but it is important to verify that it holds. We discuss methods for assessing proportionality in the next paper in this series.

### The Cox PH model fitted to the ovarian cancer data

This large database, as described in the previous paper of this series (Clark *et al*, 2003), was used to derive a prognostic index for overall survival among ovarian cancer patients in Clark *et al* (2001). Their analysis included 10 variables, but for simplicity we will consider five, all of which were measured at diagnosis: FIGO stage (an ordinal covariate taking values of 1, 2 3 or 4), histology (one of seven subtypes), grade (1, 2 or 3), ascites (yes/no) and patient age.

Table 1

**Table 1:** Hazard ratios from the Cox PH model for the ovarian dataset

	**Univariate analysis**				**Multivariate analysis**			
**Covariate**	**Coefficient (*b*_*i*_)**	**HR [exp(*b*_*i*_)]**	**95% CI**	***P*-value**	**Coefficient (*b*_*i*_)**	**HR [exp(*b*_*i*_)]**	**95% CI**	***P*-value**
FIGO stage	0.809	2.24	(2.03–2.48)	<0.001	0.731	2.08	(1.82–2.37)	<0.001
								
*Histology*				<0.001				<0.001
Serous	(0.000)	(1.00)			(0.000)	(1.00)		
Mucinous	−0.727	0.48	(0.38–0.61)		−0.422	0.66	(0.50–0.85)	
Endometroid	−1.162	0.31	(0.22–0.45)		0.198	1.22	(0.80–1.85)	
Clear cell	−0.343	0.71	(0.52–0.97)		0.342	1.41	(0.99–2.00)	
Adenocarcinoma	0.119	1.13	(0.74–1.72)		0.501	1.65	(0.91–2.99)	
Undifferentiated	0.390	1.48	(0.81–2.70)		0.746	2.11	(1.03–4.29)	
Mixed mesodermal	0.614	1.85	(1.28–2.66)		0.789	2.20	(1.45–3.35)	
								
*Grade*				<0.001				<0.001
1	(0.000)	(1.00)			(0.000)	(1.00)		
2	1.116	3.05	(1.90–4.91)		0.885	2.42	(1.40–4.19)	
3	1.650	5.20	(3.31–8.18)		0.885	2.42	(1.40–4.18)	
								
Absence of ascites	−0.798	0.45	(0.37–0.55)	<0.001	−0.396	0.67	(0.54–0.84)	<0.001
Age (per 5-year increase)	0.153	1.17	(1.12–1.21)	<0.001	0.133	1.14	(1.09–1.19)	<0.001

HR=hazard ratio, CI=confidence interval.

shows the effect sizes (given as hazard ratios), 95% confidence intervals (CI), regression coefficients and statistical significance for each of these in relation to overall survival. Each factor is assessed through separate univariate Cox regressions (left-hand columns). However, the aim of the database is to describe how the factors jointly impact on survival, and so all five factors were incorporated into the multivariate model (right-hand columns). It may be seen that higher FIGO stage, higher grade, presence of ascites and increased age impaired survival to varying (and statistically significant) degrees. The histology was also of importance: the figures describe the survival of patients with each histology type in comparison with the serous type. In principle, any type with a reasonable number of patients could be chosen as the baseline of comparison. On multivariate analysis Mucinous and serous were the tumour types with the best prognosis, whereas undifferentiated and mixed mesodermal were the worst. It is possible to present *P*-values for the comparisons between each type and serous, but we have given an overall likelihood ratio test for the differences between the categories as a whole. The FIGO stage could be modelled as a categorical variable in the same manner as grade and histology, but assuming it is a continuous variable with a linear trend across the four categories performed sufficiently well.

## PARAMETRIC PH MODELS

Parametric PH models are a class of models similar in concept and interpretation to the Cox (PH) model. The key difference between the two is that the hazard is assumed to follow a specific statistical distribution when a fully parametric PH model is fitted to the data, whereas the Cox model enforces no such constraint. Other than this, the two model types are equivalent. Hazard ratios have the same interpretation, whether derived from a Cox or a fully parametric regression model, and the proportionality of hazards is still assumed.

A number of different parametric PH models may be derived by choosing different hazard functions. As shown previously, there is a direct link between the survival and hazard, and the choice of hazard distribution determines that of the survival. In fact, the models commonly applied, such as the *Exponential*, *Weibull* or *Gompertz* models, take their names from the distribution that the survival times are assumed to follow, but the most distinguishing features between them are in the hazard function. Examples of survival and hazard functions derived from some of these parametric models were presented in the previous paper (Clark *et al*, 2003). Figure 1 shows increasing and decreasing Weibull hazard functions, as well as two groups with the latter that are proportional to each other.

### Parametric models fitted to the ovarian cancer data

The estimated hazard function of the ovarian cancer data as displayed in the previous paper (Clark *et al*, 2003) may be consistent with that derived from a Weibull PH model with decreasing hazard. Fitting this to the ovarian cancer database gives similar results as the Cox model (see Table 2

**Table 2:** Hazard ratios from the Weibull PH model for the ovarian dataset

	**Univariate analysis**				**Multivariate analysis**			
**Covariate**	**Coefficient (*b*_*i*_)**	**HR [exp(*b*_*i*_)]**	**95% CI**	***P*-value**	**Coefficient (*b*_*i*_)**	**HR [exp(*b*_*i*_)]**	**95% CI**	***P*-value**
FIGO stage	0.862	2.37	(2.14–2.62)	<0.001	0.768	2.16	(1.89–2.46)	<0.001
								
*Histology*				<0.001				<0.001
Serous	(0.000)	(1.00)			(0.000)	(1.00)		
Mucinous	−0.804	0.45	(0.35–0.57)		−0.496	0.61	(0.47–0.79)	
Endometroid	−1.276	0.28	(0.20–0.40)		0.120	1.13	(0.75–1.70)	
Clear cell	−0.419	0.66	(0.48–0.90)		0.346	1.41	(0.99–2.02)	
Adenocarcinoma	0.113	1.12	(0.73–1.71)		0.499	1.65	(0.91–2.97)	
Undifferentiated	0.397	1.49	(0.82–2.71)		0.765	2.15	(1.06–4.37)	
Mixed Mesodermal	0.638	1.89	(1.31–2.73)		0.804	2.23	(1.47–3.40)	
								
*Grade*								<0.001
1	(0.000)	(1.00)		<0.001	(0.000)	(1.00)		
2	1.154	3.17	(1.97–5.10)		0.928	2.53	(1.47–4.36)	
3	1.727	5.62	(3.58–8.84)		0.895	2.45	(1.43–4.20)	
								
Absence of ascites	−0.840	0.43	(0.36–0.52)	<0.001	−0.404	0.67	(0.54–0.83)	<0.001
Age (per 5-year increase)	0.165	1.18	(1.14–1.22)	<0.001	0.138	1.15	(1.10–1.20)	<0.001

HR=hazard ratio, CI=confidence interval.

), and may be interpreted in the same manner. Methods to check for the appropriateness of the Weibull distribution will be discussed in the next paper of this series.

## COMPARISON OF THE TWO PH APPROACHES

The main drawback of parametric models is the need to specify the distribution that most appropriately mirrors that of the actual survival times. This is an important requirement that needs to be verified and an appropriate distribution may be difficult to identify. Where a suitable distribution can be found, however, the parametric model is more informative than the Cox model. It is straightforward to derive the hazard function and to obtain predicted survival times when using a parametric model, which is not the case in the Cox framework (the use of such quantities is discussed in the next section). Additionally, the appropriate use of these models offers the advantage of being slightly more *efficient*; they yield more precise estimates (i.e. smaller standard errors).

The results from the Cox or parametric PH models may be compared directly, as the model types are merely different approaches to assessing the same quantity. For either method to be valid: (a) the covariate effect needs to be at least approximately constant throughout the duration of the study, and (b) the proportionality assumption must hold. These important issues will be addressed in the subsequent paper in this series.

## INTERPRETING THE PH MODEL: BEYOND THE HAZARD RATIO

In addition to the ratio of two hazards, it is possible to obtain other information from a PH regression model. One simple (and possibly underused) quantity that may be derived from a survival model is the predicted survival proportion at any given point in time for a particular risk group. The survival proportion for a given risk group at any time, *S*(*t*), is equal to

**Figure 2:**
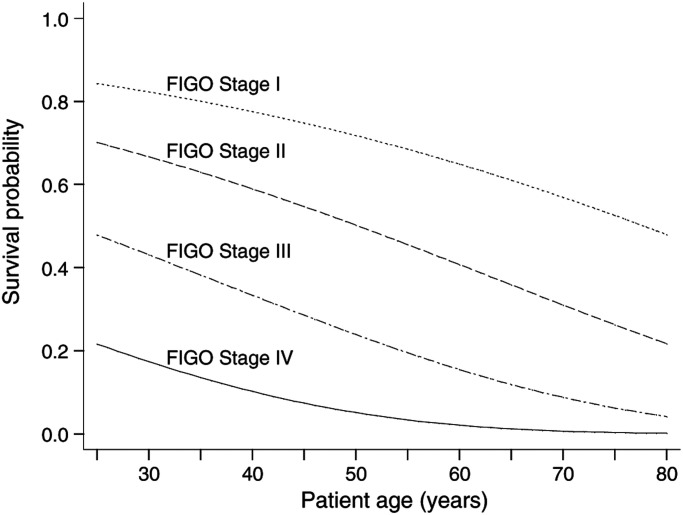
Predicted 5-year survival of ovarian cancer patients by age and FIGO stage.

*S*(*t*)=*S*_0_(*t*)^exp (*γ*)^

where *S*_0_(*t*) is the baseline survival (the survival proportion when all covariates are equal to zero) and *γ* is equal to *b*_1_*x*_1_+*b*_2_*x*_2_+⋯+*b*_*p*_*x*_*p*_. Once the value of the baseline survival at a given time is derived, then the predicted survival probabilities for patients with any specified covariate values *x_i_* are easily obtained. This information could then be displayed via tabular or graphical displays. Figure 2

illustrates this by giving predicted 5-year survival according to patient age and FIGO stage. Further examples are demonstrated by Christensen (1987) based on the Cox model, but can also be used when fitting fully parametric models. In a previous analysis that involved some of the patients in the present data, Clark *et al* (2001) produced a nomogram to summarise the impact of these and other covariates, and thus allows the reader to predict the median survival and the 2- and 5-year survival probabilities for patients with given prognostic information.

The advantage of fitting a parametric survival model is that predictions of the event survival, event hazard, mean and median survival times are readily available. For FIGO stages I–IV, the median survival times are estimated to be 7.8, 4.0, 2.0 and 1.0 years, respectively.

## ACCELERATED FAILURE TIME MODELS

The accelerated failure time (AFT) model is a different type of model that may be used for the analysis of survival time data. For a group of patients with covariates (*x*_1_, *x*_2_, … *x_p_*), the model is written mathematically as

*S*(*t*)=*S*_0_(*ϕt*)

where *S*_0_(*t*) is the baseline survivor function and *ϕ* is an ‘acceleration factor’ that depends on the covariates according to the formula

*ϕ*=exp{(*b*_1_*x*_1_+*b*_2_*x*_2_+⋯+*b*_*p*_*x*_*p*_)}.

**Figure 3:**
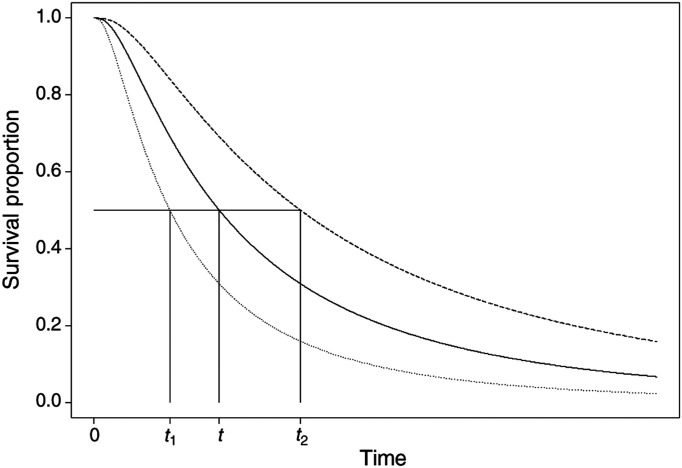
Illustration of the AFT model: (——), *S*_0_(*t*) the baseline survival function; (·······), *S*(*t_1_*)=*S_0_*(*ϕt*) for *ϕ*<1; (- - - - - -), *S*(*t*_2_)=*S*(*ϕt*) for *ϕ*>1.

The principle here is that the effect of a covariate is to stretch or shrink the survival curve along the time axis by a constant relative amount *ϕ*. Figure 3

demonstrates this for the case of a single covariate (*x*_1_) with two levels, for example, *x*_1_=0 for a placebo group and *x*_1_=1 for a new treatment group. The survival probabilities, *S*(*t*), for the placebo and new treatment groups are *S*_0_(*t*) and *S*_0_(*ϕt*), respectively. The proportion of patients who are event-free in the placebo group at any time point *t*_1_ is the same as the proportion of those who are event-free in the new treatment group at a time *t*_2_=*ϕt*_1_. Figure 3 shows the cases where *ϕ*>1 and *ϕ*<1, which represent situations where the length of survival is increased and decreased in the new treatment group compared with the placebo, respectively.

The AFT model is commonly rewritten as being log-linear with respect to time, giving

log(*T*)=*b*_0_+*b*_1_*x*_1_+*b*_2_*x*_2_+⋯+*b*_*p*_*x*_*p*_+*ɛ*

where *ɛ* is a measure of (residual) variability in the survival times. Thus, the survival times can be seen to be multiplied by a constant effect under this model specification, and the exponentiated coefficients, exp(*b_i_*), are referred to as *time ratios*. A time ratio above 1 for the covariate implies that this ‘slows down’, or prolongs the time to the event, while a time ratio below 1 indicates that an earlier event is more likely.

When the survival times follow a Weibull distribution, it can be shown that the AFT and PH models are the same. However, the AFT family of models differs crucially from the PH model types in terms of their interpretation of effect sizes as time ratios as opposed to hazard ratios.

The survival times are usually assumed to follow a specific distributional form in the AFT framework. Distributions such as the *Log-Normal, Log-Logistic, Generalised Gamma* and *Weibull* may be used to represent such survival data. Alternative methods include the method of Buckley and James (1979), which is discussed by Stare *et al* (2000), and semiparametric AFT models, in which the baseline survivor function is estimated nonparametrically (see Wei, 1992, for an overview), but have not yet been widely implemented in statistical software.

As with the PH approach, other quantities such as projected survival probabilities may be derived. Also in keeping with PH models is the fact that AFT models make assumptions; the appropriate choice of statistical distribution needs to be made, and also the covariate effects are assumed to be constant and multiplicative on the timescale, that is, that the covariate impacts on survival by a constant factor.

### Parametric AFT models fitted to the lung cancer trial data

We use the non-small cell lung cancer dataset to illustrate the AFT model, focusing on the relapse-free survival (i.e., the time from diagnosis to the reappearance of cancer, with patients censored at time of death if no recurrence had appeared). Again, we present both the univariate and multivariate effect sizes in Table 3

**Table 3:** Time ratios from the generalised gamma AFT model for the lung cancer trial

	**Univariate analysis**				**Multivariate analysis**			
**Covariate**	**Coefficient (*b*_*i*_)**	**TR exp(*b*_*i*_)**	**95% CI**	***P*-value**	**Coefficient (*b*_*i*_)**	**TR exp(*b*_*i*_)**	**95% CI**	***P*-value**
Treatment (RT+CAP *vs* RT alone)	0.648	1.91	(1.21–3.01)	0.005	0.718	2.05	(1.29–3.23)	0.002
Cell type (Sq *vs* non-Sq)	0.506	1.66	(1.01–2.71)	0.04	0.511	1.67	(1.04–2.68)	0.03
Performance status (8–10 *vs* 5–7)	0.767	2.15	(1.11–4.19)	0.02	0.729	2.07	(1.00–4.29)	0.05
*Tumour status*				0.59				0.60
1	(0.000)	(1.00)	—		(0.000)	(1.00)	—	
2	−0.189	0.83	(0.40–1.70)		−0.353	0.70	(0.35–1.41)	
3	−0.388	0.68	(0.31–1.48)		−0.378	0.69	(0.31–1.53)	
*Nodal involvement*				0.87				0.97
None	(0.000)	(1.00)	—		(0.000)	(1.00)	—	
Limited	0.122	1.13	(0.46–2.79)		−0.059	0.94	(0.36–2.48)	
Extensive	0.206	1.23	(0.55–2.73)		0.029	1.03	(0.42–2.54)	
Age at diagnosis (/years)	−0.013	0.99	(0.96–1.01)	0.34	−0.011	0.99	(0.96–1.01)	0.41
Gender (male *vs* female)	0.032	1.03	(0.62–1.71)	0.90	−0.007	0.99	(0.59–1.67)	0.98
Weight loss (⩾10 *vs* <10%)	−0.477	0.62	(0.29–1.33)	0.22	−0.337	0.71	(0.34–1.51)	0.38
Race (white *vs* non-white)	0.440	1.55	(0.81–2.98)	0.19	0.202	1.22	(0.61–2.46)	0.57

TR=time ratio, CI=confidence interval, RT=radiotherapy, CAP=cytoxan, doxorubicin and platinum-based chemotherapy, Sq=squamous.

. The specific comparison of interest was the effect of adjuvant (platinum-based) chemotherapy and radiotherapy compared with radiotherapy alone. The unadjusted treatment effect may be summarised by a time ratio of 1.91 (95% CI: 1.21–3.01; *P*=0.005), which, having allowed for other covariates increased slightly to 2.05. Therefore, we can conclude that the time to recurrence was significantly prolonged (approximately doubled) among patents given adjuvant chemotherapy in comparison with those who were not.

Again, we can derive model-based predictions: overall, patients allocated to receive adjuvant chemotherapy had a predicted median survival time of approximately 16 months, as opposed to 8 months among those treated with radiotherapy alone. Other factors are also significant and would influence these times, but these are of less importance in the context of the comparative trial. We will return to this example in the next paper of this series.

## WHICH MODEL SHOULD WE USE: PH OR AFT?

From a statistical viewpoint, an obvious way to choose between the two model types is to fit a type that is in keeping with the data. If the AFT model clearly fits the data better than the PH model, or *vice versa*, this model may be adopted as being the more appropriate. However, in some cases, either type of model may appear to fit the data adequately. In such instances, the choice of model may be influenced by other factors. For instance, if other studies of a similar nature had all used the Cox regression and reported the results as hazard ratios, one may be tempted to follow suit to aid comparability. Against this, the parametric approach offers more in the way of predictions, and the AFT formulation allows the derivation of a time ratio, which is arguably more interpretable than a ratio of two hazards. As yet, however, AFT models are relatively unfamiliar and seen rarely in medical research papers (see Kay and Kinnersley, 2002).

## OTHER APPROACHES

### Stratified survival analysis

A more straightforward way to incorporate covariates into a survival analysis is to use a stratified survival analysis. For example, suppose the covariate of primary interest is treatment, but we wish to control for the clinical stage of the tumour when making the comparison. Here, the survival in each treatment group can be compared within each stage of disease (the ‘strata’) by the logrank or some other method, and the differences within each stratum are then combined to give an overall comparison of treatments that has been adjusted for the stage.

The strength of this method is in its simplicity: as the logrank test is nonparametric, few distributional assumptions are made, and its interpretation is straightforward. Its main limitation is that it is only applicable when the covariate is categorical (or with continuous variables that have been arbitrarily categorised). Further, this method does not perform well with several covariates, as the number of individuals in each stratum quickly becomes too small to allow reasonable comparisons. In addition, it does not quantify the strength of effect of each variable, or even offer a *P*-value for factors other than the one of primary interest. This method is not generally regarded as a formal statistical model, but is of use where a very small number of covariates are to be considered, if only as an exploratory method of analysis.

### Aalen's additive model

Another approach to modelling the relationship between survival and covariates is to assume that the covariates act additively on the hazard. Aalen's additive hazard model (Aalen, 1989) is one method that has been suggested for this, but its properties are rather unlike any other model described in this paper. The covariates are assumed to impact additively upon a (unknown) baseline hazard, but the effects are not constrained to be constant. The impact is therefore allowed to vary freely over time according to the underlying equation

*h*(*t*)=*h*_0_(*t*)+*b*_1_(*t*)*x*_1_+*b*_2_(*t*)*x*_2_+⋯+*b*_*p*_(*t*)*x*_*p*_

where *h*(*t*) is the hazard, *h*_0_(*t*) is the baseline hazard and the *b_i_*(*t*) are coefficients, which may change in magnitude and even sign with time. Compare this with the Cox regression, where *h_0_*(*t*) is also estimated nonparametrically, but the *b_i_* quantify the *multiplicative* effect of covariate *i* on the hazard and are assumed constant at all times.

As it is not straightforward to estimate *h*_0_(*t*) nonparametrically, the cumulative baseline hazard is used and the regression coefficients that are actually estimated from the data are also the cumulative (additional) hazard


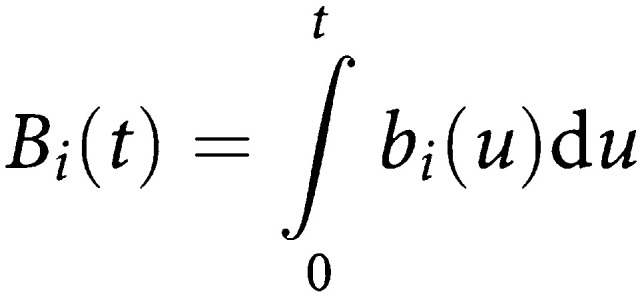


The usual method of representing these effects is to graph them against time. The further *B_i_*(*t*) is from zero at time *t*, the greater the effect the covariate has had on the hazard over the course of the study up to *t*. The values of *b_i_*(*t*), the absolute increase in hazard at time *t*, are not actually observed, but their relative size may be inferred from the slope of the line. These plots are sometimes called Aalen plots, and they are also used to provide an informal assessment of the adequacy of the proportional hazards assumption in the Cox model, although Aalen considered its primary role as an alternative model in its own right (Aalen, 1993).

The flexibility of this approach is tempered by the lack of an easy interpretation. The *B_i_(t)* coefficients are not easy to understand, and as they change repeatedly over time, can offer no single quantifiable effect size. Formal tests of statistically significant covariate effects may be carried out, but Aalen plots are essentially the only manner with which to interpret the effect sizes. These reasons, together with the relative lack of statistical software, are probably the deciding factors in the relatively minimal use of Aalen's model.

### Classification trees and artificial neural networks

Two relatively recent developments are classification trees and artificial neural networks. These methods differ substantially in their complexity and interpretation to the methods presented here and to each other. Both approaches are described in more detail in a later paper of this series.

## DISCUSSION

The principal strength of statistical models is their ability to assess several covariates simultaneously. The strengths of the stratified logrank test and other such methods are their obvious simplicity and the fact that they make fewer parametric assumptions of the data. Although these reasons are usually insufficient to suggest that the stratified method be used more widely, this second feature is a relevant one, because it needs to be kept in mind that all the models introduced here make certain distributional assumptions of the survival times that will not always be met.

We have focused on the Cox model, the class of parametric PH models and AFT models as tools with which to analyse survival time data. Other models exist (see, e.g., Collett (1994) for a more practical demonstration of some alternatives and Bagdonavičius and Nikulin (2001) for the theoretical background), but many are similar to, if not extensions of, the approaches we have discussed. The use of the Cox model offers greater flexibility than parametric alternatives and, in particular, does not require the direct estimation of the baseline hazard function (i.e. it avoids the need to specify the distribution of the survival times). However, the assumption of proportional hazards is a crucial one that needs to be fulfilled for the results to be meaningful, and will not always be satisfied. Further, while the Cox PH model may be valid, other parametric models will produce more precise estimates where the distribution is specified correctly.

A further concern is that the choice of covariates to include is also far from simple. In the third paper of this series, we will consider ways to choose between the various model types, to identify and assess the importance of covariates, and to verify that the final model is adequate.
